# A conditional random field based feature learning framework for battery capacity prediction

**DOI:** 10.1038/s41598-022-17455-x

**Published:** 2022-08-02

**Authors:** Hai-Kun Wang, Yang Zhang, Mohong Huang

**Affiliations:** 1 School of Artificial Intelligence, Chongqing University of Technology, Chongqing, 40400 China; 2 Chongqing Industrial Big Data Innovation Center Co., Ltd., Chongqing, 40400 China

**Keywords:** Computational science, Batteries

## Abstract

This paper proposes a network model framework based on long and short-term memory (LSTM) and conditional random field (CRF) to promote Li-ion battery capacity prediction results. The model uses LSTM to extract temporal features from the data and CRF to build a transfer matrix to enhance temporal feature learning for long serialization prediction of lithium battery feature sequence data. The NASA PCOE lithium battery dataset is selected for the experiments, and control tests on LSTM temporal feature extraction modules, including recurrent neural network (RNN), gated recurrent unit (GRU), bi-directional gated recurrent unit (BiGRU) and bi-directional long and short term memory (BiLSTM) networks, are designed to test the adaptability of the CRF method to different temporal feature extraction modules. Compared with previous Li-ion battery capacity prediction methods, the network model framework proposed in this paper achieves better prediction results in terms of root mean square error (RMSE) and mean absolute percentage error (MAPE) metrics.

## Introduction

Rechargeable lithium-ion batteries play a crucial role in many modern applications^1,2^, from portable electronics and medical devices to renewable energy integration in power grids and electric vehicles, due to their long cycle life, low self-discharge rate, wide range of applications, and low environmental pollution^3^. However, as a product of industrialization, the performance of lithium batteries^4^ decreases with increasing usage time. As the capacity of the battery decreases, the battery will eventually fail. Therefore, it is important to predict the future health^5^ of lithium batteries to improve the reliability of energy systems.

The future health of Li-ion batteries is usually measured by the indicator state of health (SOH), which reflects the battery's usage by calculating the decay process of the battery capacity and obtaining the difference between the battery's current usage state and its factory characteristics. When the current capacity decreases from 80 to 70% of the nominal capacity, the performance of the battery will decline rapidly. When the capacity of a lithium battery decreases to 70%, it is usually regarded as a battery failure. Therefore the accuracy of battery capacity prediction often affects the SOH calculation results, leading to bias in the estimation of battery usage and affecting the subsequent predictive maintenance strategy and energy system management. Li-ion battery capacity prediction^6^ is essentially a regression problem. Current methods for capacity decay estimation mainly include equivalent circuit models, electrochemical models and data-driven models^7^.

The equivalent circuit model uses circuit elements with empirical nonlinear parameters to build a mathematical model representing the battery system and combines measured data to estimate battery capacity and SOH^8,9^. Wei et al.^10^ used the Thevenin model by constructing the battery health index online, using the health index as the input parameter and the battery capacity decay as the output parameter in a gray neural network model to estimate the battery SOH. He et al.^11^ compared resistor-circuit equivalent circuit models of different orders, and adding RC series modules using the high nonlinearity of the battery operating characteristics can improve the accuracy and reliability of the model prediction, but reduces the applicability of real-time^12^. The capacity prediction method of the equivalent circuit model has the advantages of fewer parameters and high timeliness, but it is difficult to achieve higher prediction accuracy because it ignores the effects caused by environmental changes and data acquisition during the operation of lithium batteries^13,14^.

The electrochemical model establishes a physical model for SOH estimation and prediction by studying the chemical processes occurring inside the battery during operation^15^. Zhang et al.^16^ improved the prediction accuracy by analyzing the impedance characteristics based on the changes in the impedance properties of the battery. Ashwin et al.^17^ established an electrochemical battery aging model under cyclic loading conditions and constructed the capacity decay of the lithium-ion battery process. The electrochemical modeling method can accurately describe the movement pattern of positive and negative electrodes and the changing trend of active substances during the capacity decay of lithium ions by analyzing the detailed internal electrochemical reaction process and reaction intensity during the aging process of the battery. However, the electrochemical system is more complex and the characteristic parameters are coupled with each other, which makes its dynamic prediction accuracy poor and difficult to achieve wide applicability.

The data-driven model establishes the mapping relationship between the characteristic parameters and the health condition from the overall level of the data by extracting the characteristic values of the measured parameters^18,19^. Depending on the data mining methods, they are mainly divided into statistical filtering methods, support vector methods, neural network methods, and fusion methods. Statistical filtering is a method to extract and reproduce valid signals and waveforms from data containing a large number of noisy signals, and the best weighting factor with a strong target following ability is automatically calculated and determined by a recursive linear data processing algorithm^20,21^. He et al.^22^ used the extended Kalman filtering (EKF) algorithm to estimate the unknown parameters in the time degradation parameter model of lithium-ion battery capacity and obtained the future phase prediction results of the degradation trend of the remaining battery capacity. Support vector machine (SVM) as a nonlinear data analysis method, can not only provide relatively accurate estimation and prediction results with a small amount of data but also improve the data quality to a certain extent and overcome the drawback that the model falls into the optimal local extremes. Fewer unknown parameters and high sparsity are the characteristics of this method^23,24^. Zhang et al.^25^ used to improve the prediction performance and operational efficiency of the battery by optimizing the relevance vector machine (RVM), by optimizing the RVM. Gao et al.^26^ proposed a single radial basis kernel function based on the novel multicore SVM based on polynomial kernel and radial basis kernel function for predicting the remaining useful life (RUL) of Li-ion batteries, which has better prediction accuracy and stronger generalization ability compared to SVM while reducing training time and computational complexity. A neural network is a nonlinear prediction method composed of many neurons according to certain rules. The neurons contained in the network model are trained to connect weights and thresholds to build an accurate estimation and prediction model^27,28^. Increasing the depth of the neural network can approach any nonlinear mapping with a simple structure and high learning ability^29,30^. Neural network approaches mainly include artificial neural networks (ANN), convolutional neural networks (CNN), back propagation neural networks (BP), gated recurrent units (GRU), and long short-term memory networks (LSTM). Zhang et al.^31^ used LSTM and RNN networks to capture the long-standing relationship between lithium battery capacity degradation for prediction. Fan et al.^32^ proposed a GRU-CNN network for learning shared information and time dependence of charging profiles, including characteristic variation profiles such as voltage, current, and temperature, for estimating SOH. Zhou et al.^33^ improved the prediction accuracy of the model by capturing the local capacity regeneration phenomenon generated by the battery during charging and discharging through time convolutional networks (TCN). The fusion method is based on the characteristics of different algorithms, each taking their strengths for fusion improvement, which not only ensures the accuracy of the predicted data but also provides an accurate assessment of the prediction uncertainty. Liu et al.^34^ proposed a fusion algorithm based on least squares support vector regression (LSSVR) and hidden markov model (HMM) to predict the health status of rolling bearings, where LSSVR was used to predict the feature signal, and HMM was used to identify state features. Hong et al.^35^ proposed a fusion estimation method for SOH of lithium-ion batteries based on capacity incremental analysis and a weighted Kalman filter algorithm, which has higher prediction accuracy compared to the common Kalman filter method. The recent Li-ion battery capacity prediction models are detailed in Table 1.

**Table 1:** Recent prediction models for lithium battery.

Authors	Year	Approach
Zhang et al.^31^	2016	Relevance vector machine
Wang et al.^37^	2017	State space model
Gao et al.^26^	2017	Multi-kernel support vector machine with particle swarm optimization
Zhang et al.^38^	2018	Particle filter and unscented Kalman filter
Zhang et al.^25^	2018	LTSM
Ren et al.^41^	2018	Autoencoder with deep neural network
Fang et al.^36^	2019	Double extended Kalman filter
Deng et al.^43^	2019	Least squares support vector machine
Fan et al.^32^	2020	GRU-CNN
Zhou et al. ^33^	2020	Temporal convolutional network
Song et al.^39^	2020	Principal component analysis and support vector machine
Ren et al.^40^	2020	CNN-LSTM
Kodjo S.R.Mawonou et al.^42^	2021	Random forest
Hong et al.^44^	2021	Locally linear embedding and isomap
Jungsoo Kim et al.^45^	2022	Genetic algorithm and pseudo-2-dimensional model

To improve the accuracy of lithium battery capacity prediction, this paper proposes a Li-battery capacity prediction model with CRF as the core. CRF is a discriminative probabilistic model about the temporal sequence, which is widely used in natural language processing (NLP)^46,47^. CRF constructs the state transfer matrix by the trend of the changing relationship of the neighboring labels and obtains the probability distribution of the prediction sequence by reverse decoding, where the state sequence with the highest probability is the optimal prediction result. The model adds CNN networks to learn feature data at different scales and LSTM networks to collect time-series relationship information. The feasibility and effectiveness of the model were verified on the lithium battery dataset provided by NASA, and the prediction accuracy of the model was improved compared with other network models. The CNN-LSTM-CRF model provides a new idea for the lithium battery prediction problem.

The main contributions of this work are:

1. The CRF method is attempted to be introduced in the capacity prediction problem to calculate the observed state of the capacity prediction sequence by the offset matrix of the feature data, which more intuitively reflects the change of the capacity decline trend.
2. To improve the prediction accuracy of the CRF model, the study incorporates a CNN convolution module for collecting feature data at different time scales and an RNN time-linked module for capturing the changing trend of feature data on the before-and-after time difference and extracting its time-series relationship information. To verify the fit of the CRF prediction model to different time-linked modules, the study added GRU, LSTM, BiLSTM, and other control experiments, and the experimental results on the NASA lithium battery dataset showed that LSTM achieved better results.

## Methodology

### Overall framework of model

Lithium-ion battery residual life prediction is based on the analysis and processing of lithium battery use data to estimate the residual life of the battery. This paper studies how to make the prediction results more accurate and improve the robustness of the model.

**Figure 1:**
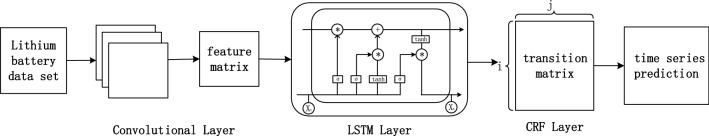
Network model framework.

Since the test time points of each charge and discharge cycle are different, the test number of one cycle at the maximum collection point in the data set is taken as the standard, and the zero vector is used to supplement the insufficient ones. The collected data is first trained through the CNN model of the convolutional window, and then the extracted feature information vector containing the timing relationship is output to the LSTM network for training. After training, a complete implicit state sequence is obtained, namely the vector containing the timing sequence feature information of the charging-discharge cycle. Because the CRF has a good effect on time-series prediction, the vector with time series feature information trained by LSTM is input into CRF model, and the final prediction result is obtained by CRF.The overall framework of the model is shown in Fig. 1.

### CNN network

**Figure 2:**
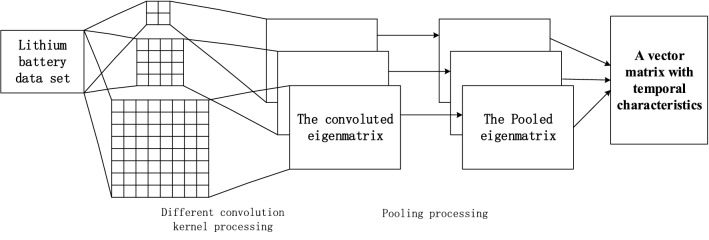
Structure of CNN network model.

The CNN module mainly uses the convolutional layer in the convolutional neural network to capture the local features of the data, and uses a variety of different convolutional cores to carry out the convolution operation. Then, the Maxpooling operation is used to further extract the most effective features of the local features, while reducing overfitting. Then, the vector of local features of battery test data containing time-series relationship obtained after convolution and pooling is fused to obtain more effective feature information The CNN model established in this paper is shown in Fig. 2.

1. Input layer: This layer is mainly used to receive the initial battery characteristic data. The feature data matrix R is obtained by two-dimensional reconstruction of multi-feature timing^49^series test data. As shown in Eq. (1), R is connected to the CNN model as the input layer matrix.
  1 $$R = m\sum\limits_{i = 1}^{n} {x_{n} } \begin{array}{*{20}c} {} & {m \in \{ t,f\} } \\ \end{array}$$
  where $m$ represents the dimension selected for construction, $t$ represents the time dimension, $f$ represents the characteristic dimension, and $x_{n}$ represents the battery data measured in the NTH charge–discharge cycle.
2. Convolutional layer: This layer can use different sizes of convolution windows to perform convolution operations. The parameters of the convolutional neural network are stored in the weight matrix and the bias matrix. The initial value is randomly generated and changed through training. Due to the difference in the size of the convolution kernel, through the convolution operation, various forms of local features can be extracted, as shown in Eq. (2).
  2 $$G = f(a*c) + b$$
  Among them, $a$ is the weight, $c$ is the convolution vector matrix to be calculated, $b$ is the bias, and $f$ selects the ReLU activation function.
  For all neurons in the next layer, they are calculated by the convolution kernel of the previous layer, so they represent the characteristics of the neurons in the previous layer detected from different positions. Since multiple convolution kernels are used in the CNN module to calculate the feature mapping matrix of the next layer, multiple feature mapping matrices $G_{w}$ of the next layer are obtained, where $w$ represents the type of convolution window size used, that is, the final CNN integrated Number.
3. Pooling layer: This layer validates the information extracted from the convolutional layer matrix through maxpooling operation to obtain multiple feature mapping matrices $P_{w}$; then the pooled multiple feature matrices are compressed into a feature matrix $\overline{P}$, this process is called It is CNN integration. The integration formula is shown in formula (3). The dimensions of the compressed matrix rows are the same as the initial input ${\text{X}}$ matrix, but the data in this matrix can express more characteristic information.
  3 $$\overline{P} = \frac{1}{m}\sum\limits_{i = 1}^{m} {P_{n} }$$

In this way, the input word vector is subjected to multi-layer convolution and pooling operations, then an eigenvector matrix containing the timing relationship is obtained, finally this matrix is used as the input of the next layer of LSTM model.

### LSTM network

**Figure 3:**
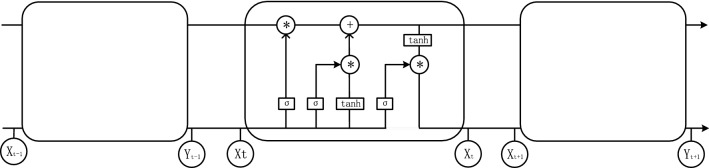
Structure of LSTM network model.

The second layer of the model is the LSTM layer, which is used to deal with timing features. The core of LSTM has a four-layer structure, which mainly contains three gates (forgetting gate, input gate, output gate) and a memory unit. The LSTM network model is shown in Fig. 3.

LSTM uses the forget gate to determine what information can pass through the state unit. The forget gate determines how much information can pass through at the previous time based on the output $h_{t - 1}$ and the current input $x_{t}$ at the previous time. The calculation of $f_{t}$ is shown in Eq. (4).

4 $$f_{t} = \sigma (W_{f} x_{t} + U_{f} h_{t - 1} + b_{f} )$$

Through the input gate to generate new information that needs to be updated. This step consists of two parts: the first part is to determine the value for updating $i_{t}$ obtained from the input gate; the second part is to use the Tanh layer to generate a new candidate value $\tilde{C}_{t}$, which is added as the candidate value generated by the current layer To the state unit. Then combine the values generated by the two parts to update.The calculations of $i_{t}$ and $\tilde{C}_{t}$ are as follows:

5 $$i_{t} = \sigma (W_{i} x_{t} + U_{i} h_{t - 1} + b_{i} )$$

6 $$\tilde{C}_{t} = \tanh (W_{{\overline{c}}} x_{t} + U_{{\overline{c}}} h_{t - 1} )$$

Combine the forget gate and input gate, that is, discard redundant information and add new information:

7 $$C_{t} = f_{t} C_{t - 1} + i_{t} \tilde{C}_{t}$$

The last step is to determine the output of the model. First, get an initial output through the sigmoid layer, and then use Tanh to scale the ct value to −1 to 1, and then multiply the output from the sigmoid to get the output of the model.

8 $$o_{t} = \sigma (W_{o} x_{t} + U_{o} h_{t - 1} + b_{o} ),h_{t} = o_{t} \tanh (C_{t} )$$

where $\sigma$ is the sigmoid activation function; tanh represents the hyperbolic tangent activation function; $W_{f} ,W_{i} ,W_{o} ,U_{f} ,U_{i} ,U_{o}$ $W_{f}$ represent the weight matrix of input gate, forget gate, and output gate respectively; $b_{f} ,b_{i} ,b_{o}$ represents the bias vector of the input gate, forget gate, and output gate; $h_{t}$ represents the output at time $t$.

### CRF network

In the prediction task, LSTM is good at processing long-term series of test data, but it cannot coordinate the dependence between adjacent results of time series data, especially in the face of battery capacity regeneration. CRF can obtain an optimal prediction result through the relationship of neighboring data, and make up for the shortcomings of LSTM. For any sequence $X = (x_{1} ,x_{2} , \cdots ,x_{n} )$, assume that $p$ is the output matrix of the LSTM, and the size of $p$ is $n*k$, where $n$ is the time series prediction step size, $k$ is the measurement feature information, and $p_{ij}$ represents the jth measurement of the i-th time point word feature. For the prediction sequence $Y = (y_{1} ,y_{2} , \cdots ,y_{n} )$, the score function to get it is:

9 $$s(X,Y) = \sum\limits_{i = 0}^{n} {A_{{y_{i} ,y_{i + 1} }} } + \sum\limits_{i = 1}^{n} {p_{{P_{i} ,y_{i} }} }$$

A represents the transition score matrix, A represents the score which the predicted value $i$ is transferred to $j$, and the probability of the predicted sequence ${\text{Y}}$ is:

10 $$p(Y\left| X \right.) = \frac{{e^{s(X,Y)} }}{{\sum\limits_{{\tilde{Y} \in Y_{X} }} {s(X,\tilde{Y})} }}$$

Take the logarithm at both ends to get the likelihood function of the predicted sequence:

11 $$\ln (p(Y\left| X \right.)) = s(X,Y) - \ln (\sum\limits_{{\tilde{Y} \in Y_{X} }} {s(X,\tilde{Y})} )$$

In the formula, ${\tilde{\text{Y}}}$ represents the real labeling sequence, and ${\text{Y}}_{{\text{X}}}$ represents all possible labeling sequences. The output sequence with the largest score after decoding:

12 $$Y^{*} = \mathop {\arg \max }\limits_{{\tilde{Y} \in Y_{X} }} s(X,\tilde{Y})$$

**Figure 4:**
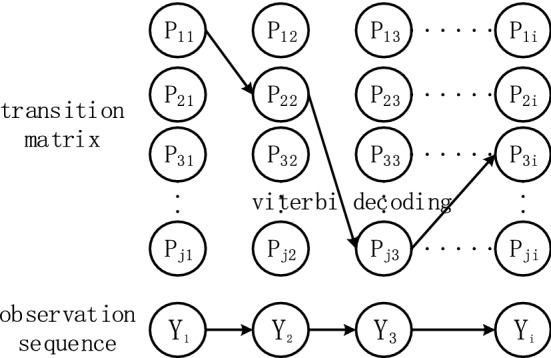
Structure of CRF model.

The CRF model is shown in Fig. 4.

## Experiment

### Description of lithium-ion battery datasets

**Figure 5:**
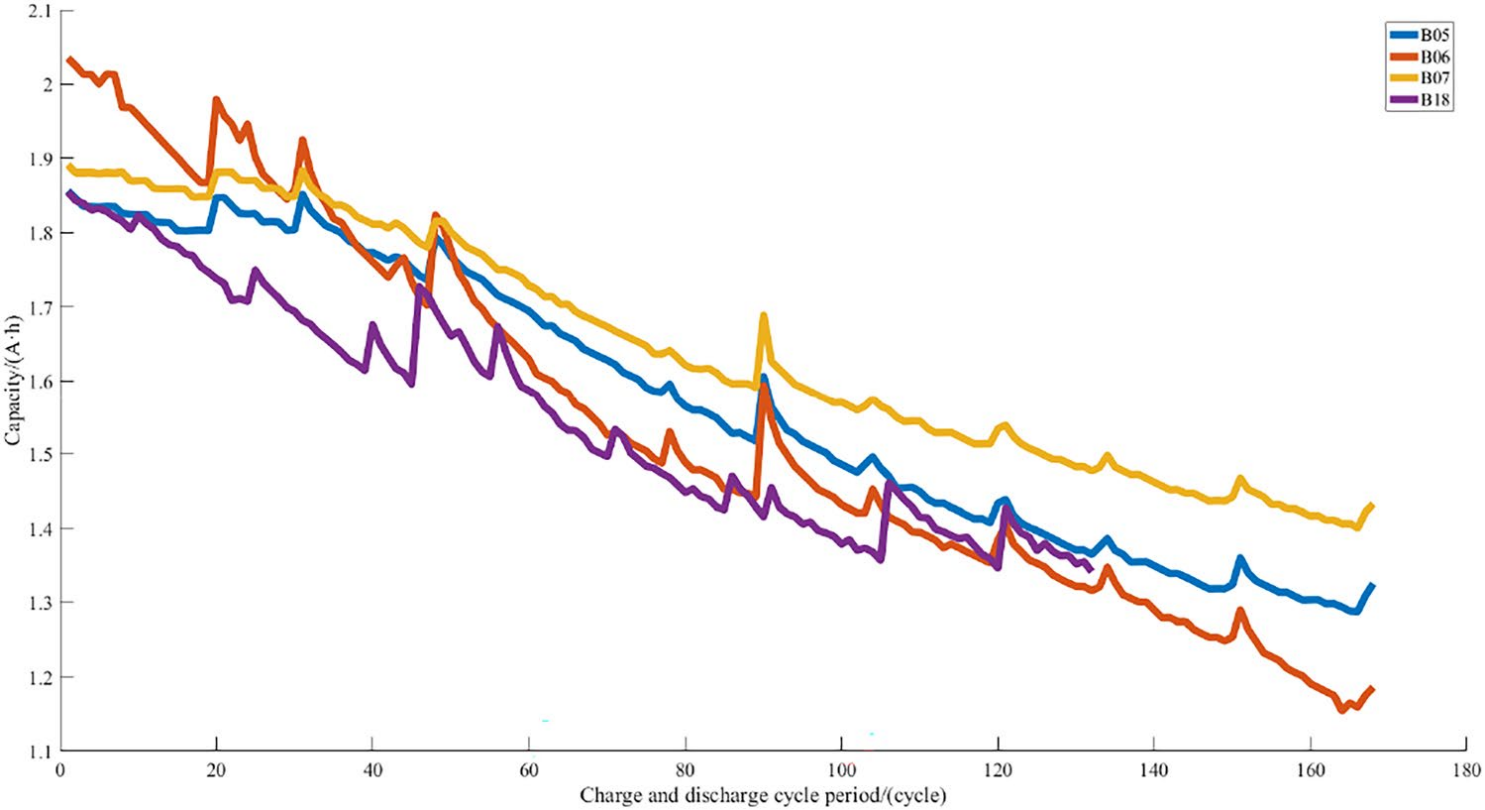
Chart of battery capacity decline.

The data used in the experiment came from the NASA PCOE lithium-ion battery data set^48^. A set of four Li-ion batteries (B05, B06, B07, and B18) were run through 3 different operational profiles (charge, discharge and impedance) at room temperature. Charging was carried out in a constant current mode at 1.5A until the battery voltage reached 4.2 V and then continued in a constant voltage mode until the charge current dropped to 20 mA. Discharge was carried out at a constant current level of 2A until the battery voltage fell to 2.7 V, 2.5 V, 2.2 V, and 2.5 V for batteries B05, B06, B07, and B18 respectively. Impedance measurement was carried out through an electrochemical impedance spectroscopy frequency sweep from 0.1 Hz to 5 kHz. Repeated charge and discharge cycles result in accelerated aging of the batteries while impedance measurements provide insight into the internal battery parameters that change as aging progresses. This dataset can be used for the prediction of both the remaining charge and remaining useful life.Rom the Fig. 5 that the capacity of the battery is gradually decreasing as the charging and discharging cycle continues. The sudden increase of points in the Fig. 5 is due to the capacity regeneration effect.

The data set contains six statistical test features, namely Voltage_Measured, Current_Measured, Temperature_Measured, Current_Load, Voltage_Load and Time.

### Datasets segmentation

Normalization of data can be used in machine learning algorithms to eliminate the negative effects of different value ranges and to improve the convergence speed and accuracy of the model. The method used in this study is min–max normalization, which scales the data to the interval [0, 1] through a linear transformation:

13 $${\text{Norm}} \left( {X^{f} } \right) = \frac{{\left( {X^{f} - X_{{\min^{f} }} } \right)}}{{X_{max}^{f} - X_{{\min^{f} }} + \varepsilon }}$$

where $X^{f}$ is the all readings of sensor $f$ on all units, $\varepsilon$ denotes a positive number that tends to 0 infinitely, preventing the case where the denominator is 0.

### Datasets segmentation

**Figure 6:**
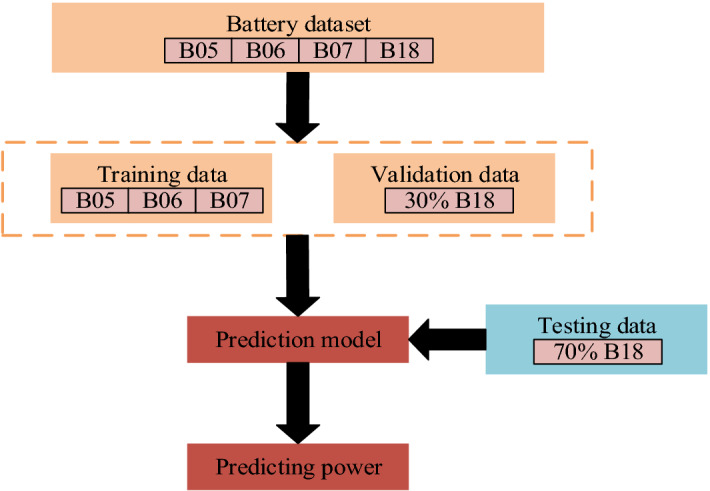
Partition of battery datasets.

In order to verify the generalizability of the prediction results of this framework, three sets of data are randomly selected from four battery datasets as the training set and another set as the validation and test set. Figure 6 details the overall process of datasets partitioning.

### Experimental configuration

The experimental code in this paper runs in Python 3.7 environment; the deep learning frameworks are Tensorflow 1.15.2 and Keras 2.2.4; the experiments are implemented on a PC (Windows 10 OS, Intel (R) Core (TM) I9-10900 KF CPU 3.7 GHz, 24 Gbytes of RAM. NVIDIA GeForce RTX 3090 GPU).

### Parameter configuration

The correct choice of network model parameters often affects the prediction results. The experiment sets the parameters of step size of the predicted time series, the number of neurons in the network layer, learning rate and batch_size as hyperparameters, and the detailed data are shown in Table 2. The ReLU activation function is selected in the convolutional layer, the linear activation function is selected in the fully connected layer, and the marginal learning mode is selected in the CRF.

**Table 2:** Hyperparametric range.

Parameter	Range
cnn_1 neurons	2–16
cnn_kernel	2–18
cnn_2 neurons	2–36
MaxPooling neurons	2–min(cnn_1,cnn_kernel,cnn_2)
RNN neurons	6–200
CRF neurons	6–200
Dense neurons	6–200
Learning rate	0.01–0.0001
Batch size	1–200

**Figure 7:**
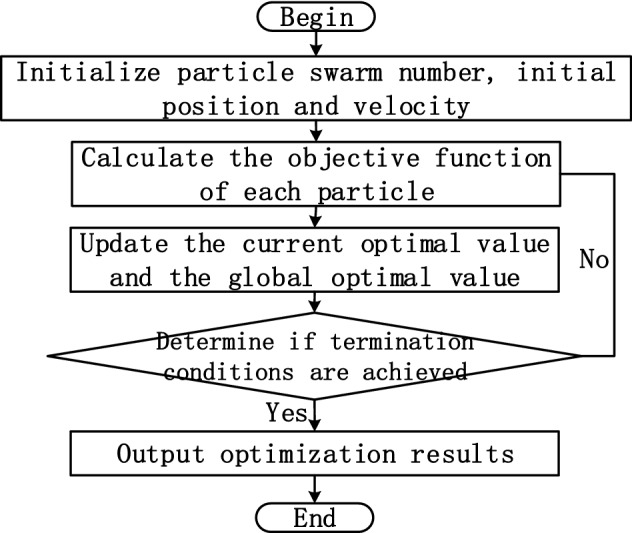
Process structure of PSO.

To obtain the hyperparameters suitable for the network model faster, particle swarm optimization (PSO) was experimentally chosen as the parameter optimization algorithm. PSO is a swarm intelligence algorithm for finding optimal parameters, which is often used in the parameter finding the process of network models in battery prediction problems^49,50^. PSO completes the search process by the individual search for optimal values and population information sharing, and the Fig. 7 shows the parameter optimization process of the particle swarm algorithm in detail.

In the experiment is divided into the following specific steps:

1. Parameter initialization. Set the number of particles $n = 10$, the particle size $D$ as the number of parameters to be optimized 10, the learning factor of particle update $c_{1} = 1$ and $c_{2} = 0.5$, the number of iterations $M = 100$, and the inertia weight parameter $w = 0.8$. Randomly generate the initial velocity information $v_{ij}$ and position information $x_{ij}$ of the particle.
2. The mean square error of the prediction result is used as the objective function of the particles, and the calculation formula is as follows.
  14 $$MSE = \frac{1}{N}\sum\limits_{{{\text{t}} = 1}}^{N} {(x^{\prime}_{t} - x_{t} )}^{2}$$
3. Calculate and update the current optimal solution $p_{i}$ and the global optimal solution $p^{\prime}_{i}$ obtained from the particle calculation of the current iteration number.
4. Update the velocity and position information of the particle, and update the formula as follows:
  15 $$v_{ij} (t + 1) = w \cdot v_{ij} (t) + c_{1} r_{1} [p_{i} (t) - x_{ij} (t)] + c_{2} r_{2} [p^{\prime}_{i} (t) - x_{ij} (t)]$$
  16 $$x_{ij} (t + 1) = x_{ij} (t) + v_{ij} (t + 1)$$
  where $r_{1}$ and $r_{2}$ is taken as a uniform random number in the range of [0–1], so that the particle swarm algorithm has the ability to search randomly to avoid falling into local optimum.
5. Judge whether the loop reaches the termination condition maximum number of iterations $M$, reaches the termination condition then end the optimization process to get the optimization result $p^{\prime}_{i}$.

The results of the PSO optimization parameters are shown in Table 3.

**Table 3:** Hyperparameter optimization results.

Parameter	Range
cnn_1 neurons	9
cnn_kernel	17
cnn_2 neurons	6
MaxPooling neurons	5
RNN neurons	192
CRF neurons	76
Dense neurons	199
Learning rate	0.0005
Batch size	2

### Evaluation metrics

To quantify the forecast results for comparison and analysis. RMSE and MAPE are used to evaluate the performance of the model in this paper^51^. The calculation methods of each evaluation index are as follows:

17 $$RMSE = \sqrt {\frac{1}{N}\sum\limits_{{{\text{t}} = 1}}^{N} {(predicted_{t} - observed_{t} )}^{2} }$$

18 $$MAPE = \sum\limits_{{{\text{t}} = 1}}^{N} {\left| {\frac{{predicted_{t} - observed_{t} }}{{observed_{t} }}} \right| \times \frac{100}{N}}$$

In the formula, $N$ is the total number of measurements predicted by the model. The results were averaged over several experiments.

## Experimental results and discussion

### Results of time-linked module control experiment

To test the effect of different temporal association modules on the prediction accuracy in CRF models, control experiments of LSTM, GRU, and BiLSTM were designed. The temporal association module compares the difference between using a single-layer CNN network to extract feature information as network input and using a two-layer CNN network. The output of the temporal association module is used as the input of the CRF model, and the experiments are done on B18, and the experimental results are shown in Table 4.

**Table 4:** CNN and RNN compared the experimental prediction results.

CNN	RNN	RMSE	MAPE
Single layer	LSTM	0.0367	0.0264
	GRU	0.0589	0.0352
	BiLSTM	0.0496	0.0314
Double layer	LSTM	0.0248	0.0141
	GRU	0.0316	0.0236
	BiLSTM	0.0421	0.0272

The experimental results surface that the prediction error of LSTM is smaller compared with other RNNs, which indicates that the temporal information extracted by LSTM is more adapted to the input with the CRF network. Compared with single-layer CNN networks, two-layer CNN networks can obtain better results. We found that this is because the two-layer CNN changes the length of the input to the network model using the pooling layer compared to the single-layer CNN, which enables the second layer CNN to extract a wider range of feature information.

### Results of CRF ablation experiments

To prove the effect of CRF on the model, the effects of adding CRF and not adding CRF on the CNN-LSTM-CRF predicted results were compared. The comparison results are shown in Table 5.

**Table 5:** CRF comparison experiment predicted the results.

Data	Cycle	CRF		Without CRF	
		RMSE	MAPE	RMSE	MAPE
B05	168	0.0216	0.0161	0.0316	0.0253
B06	168	0.0179	0.0081	0.0275	0.0152
B07	168	0.0257	0.0193	0.0357	0.0284
B18	132	0.0316	0.0230	0.0411	0.0325

The experimental results found that CRF could improve the accuracy of network model prediction, and the RMSE and MAPE evaluation metrics on four datasets B05, B06, B07, and B18 improved by more than 20% on average compared with no CRF, with the MAPE metric of B06 dataset improving by 53% as the largest improvement of the experiment, which indicated the importance of CRF model, which was also proved on the subsequent experiments of the probability distribution of prediction results.

### Results of capacity prediction

**Figure 8:**
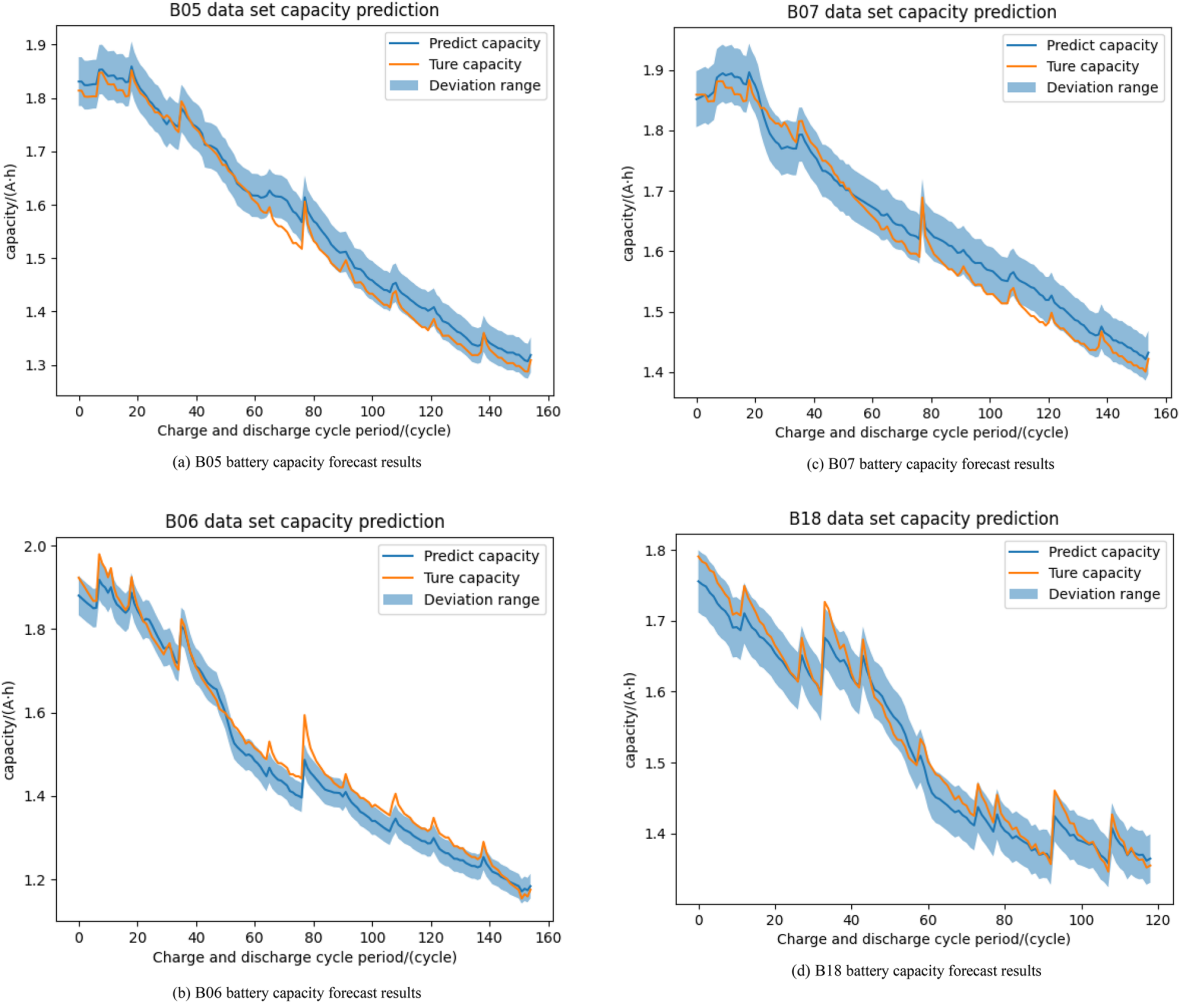
B05, B06, B07 and B18 battery capacity forecast results.

To intuitively reflect the prediction results of this method, Fig. 8 shows in detail the original measurement capacity and model prediction capacity of battery data sets B05, B06, B07, and B18(threshold value of precision region α is ± 2.5%).

The results from the figure show that most of the predicted results are within the error range of the true capacity. Of course, the predicted values at 78 cycles on the B05, B06, and B07 data sets show poorer prediction results, which is due to the capacity rebound caused by the capacity regeneration phenomenon during the charging and discharging process of Li-ion batteries, and the sudden change in capacity will bring a larger prediction error compared to the smooth state. This is because we use the complete battery dataset for model training to predict a different battery dataset, and the CRF model uses the feature offset matrix during the training process to count the overall trend of the training data and record the overall trend of the battery capacity so that when there is a large error, the error will be reduced in the subsequent prediction process according to the learning record. The error is reduced in the subsequent prediction process based on the learning records.

### Comparison of the previous model

In order to verify the prediction superiority of the CNN-LSTM-CRF model proposed in this paper, comparative experiments were conducted with SVM, LSTM, and GRU models. The RMSE and MAPE results of the four models are compared in Table 6, which can more intuitively show the prediction accuracy of this algorithm.

**Table 6:** Comparison of prediction results of 4 algorithms.

Evaluation index	Data	SVM	LSTM	GRU	CNN-LSTM-CRF
RMSE	B05	0.0809	0.0511	0.0510	0.0216
	B06	0.0804	0.0866	0.0818	0.0179
	B07	0.0679	0.0475	0.0428	0.0257
	B18	0.0731	0.0534	0.0529	0.0316
MAPE	B05	0.0567	0.0370	0.0370	0.0161
	B06	0.0606	0.0650	0.0616	0.0081
	B07	0.0447	0.0319	0.0288	0.0193
	B18	0.0526	0.0479	0.0448	0.0230

It can be found from the table that for RMSE and MAPE metrics, the average metrics of the predicted value of the model in this paper are superior to the comparison model, illustrating the feasibility of the CNN-LSTM-CRF model proposed in this paper in the battery capacity prediction problem.

## Conclusion

For the problem of lithium battery capacity prediction, this paper takes inspiration from the field of NLP and proposes a combined CNN-LSTM-CRF neural network prediction model, which is applied to the battery remaining life prediction for the first time. The model inputs continuous-time battery measurement data and predicts the output battery capacity situation at the current time point to obtain the remaining battery life at this time. Compared with the previous battery capacity prediction network model, the major difference in this model is the inclusion of CRF. The capacity prediction sequence is calculated by the offset matrix of the feature data, which more intuitively reflects the change of the decreasing trend of capacity. The CNN convolutional module is added to the model to collect the feature data, and the time-linked module captures the trend of feature data in the time dimension to extract the temporal information. Among them, LSTM achieves better results in the time-linked module control experiments. The ablation experiments demonstrate the effectiveness of the CRF network in the capacity prediction process. By comparing with previous models, our model achieves better prediction results.

Our model still has flaws. The large number of network structures combined makes the network depth and computation of the model huge, which will cost more computational resources and time. Future work can try to experiment with migration learning in the model learning process, and use the extracted trained network parameters to adjust the network model to make the real-time prediction of the model possible.

## Data Availability

NASA PCOE lithium-ion battery data used to support this study are available at https://ti.arc.nasa.gov/tech/dash/groups/pcoe/prognostic-data-repository.
